# Effect of the ceramic membrane properties on the microbial fuel cell power output and catholyte generation

**DOI:** 10.1016/j.jpowsour.2019.04.043

**Published:** 2019-07-31

**Authors:** Irene Merino-Jimenez, Fernando Gonzalez-Juarez, John Greenman, Ioannis Ieropoulos

**Affiliations:** a Bristol BioEnergy Centre, Bristol Robotics Laboratory, University of the West of England, BS16 1QY, UK; b Biological, Biomedical and Analytical Sciences, University of the West of England, BS16 1QY, UK; c Research and Development Ceramics, ROCA Sanitario, S.A., Av. de La Generalitat, 231, 08840, Barcelona, Spain

**Keywords:** Microbial fuel cell (MFC), Ceramic membrane, Catholyte production, Electroosmotic drag, Urine

## Abstract

Ceramic membranes for MFCs offer a low cost alternative to the expensive ion exchange membranes, whilst promoting catholyte accumulation. However, their physicochemical properties need to be optimised, in order to increase the power output and the catholyte quality from MFCs. Two compositions of fine fire clay (FFC) cured under three firing cycles were manufactured, analysed and tested as ion-exchange and structural material for MFCs. The samples were characterised by scanning electron microscopy (SEM) and electrochemical impedance spectroscopy (EIS). The power and catholyte generated from the ceramic MFCs with different FFC types was also evaluated. The results show a direct correlation between the ohmic resistance, the MFC power generation and the water absorption of the ceramics, giving a maximum power of 1 mW from the MFC with the most absorptive FFC (16.37% water absorbance). A slightly more alkaline catholyte was synthesised from the MFCs with higher water absorption FFC.

## Highlights

• The physico-chemical properties of ceramic membranes affect the MFC performance.
• Fine fire clay ceramics with higher absorption leads to higher MFC power output.
• The power generated increased by 64% when using more absorptive ceramic.
• More alkaline catholyte was obtained from the MFCs with more absorptive clay.
• Catholyte quality and power from the MFC enhanced by modifying the clay properties.

## Graphical abstract

**Figure:**
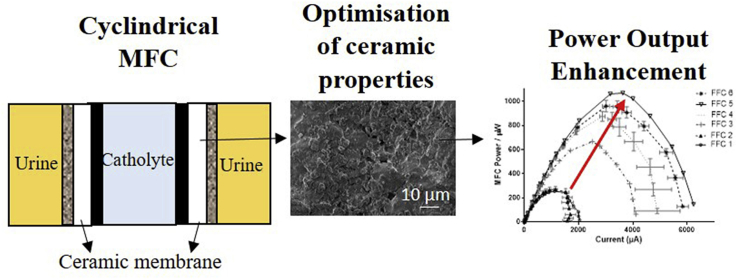

## 1 Introduction

Microbial fuel cells (MFCs) have emerged as a promising technology to address both power generation and water management, particularly in remote areas [10,16,23,38]. MFCs treating urine have reported to generate enough power to light an internal toilet, achieving a power output of ≈150 mW (19.2 L displacement volume) [13,14] and a urine treatment near the disposable EU levels (0.125 mg COD^.^ L^−1^), with a decrease of the Chemical Oxygen Demand (COD) from 5.586 mg COD^.^ L^−1^ to 0.625 mg COD^.^ L^−1^ (88%) [39]. MFCs employ electrochemically-active bacteria in the anode half-cell to generate electricity from organic molecules through their anaerobic metabolism, whilst the cathode may be biotic or abiotic. Generally, the MFC configuration is based on an anodic and a cathodic chamber separated by a semipermeable membrane. At the cathode, oxygen from the air is primarily used as the oxidant, due to its abundance and high reduction potential [35,43]. The oxygen reduction reaction (ORR) and its low kinetics remain one of the challenges of the fuel cell technology [35]. MFCs are low power generation devices and in an attempt to increase their performance, high tech components and configurations are used, such as expensive catalysts and proton exchange membranes. The use of activated carbon as a catalyst for the ORR has been generally accepted by the research community, since it offers a good enough catalytic activity at an affordable cost and longevity [30,37,42]. However, MFC applications are emerging as a promising technology for remote locations, developing countries or emergency areas, including refugee camps, where the wastewater could be treated whilst electricity is generated. In order for the MFCs to reach commercialisation and real-life practical implementation, affordable and effective materials need to be used. Following this approach, the use of ceramics as a membrane and supporting material for microbial fuel cells has gained interest in recent years [1,40,41]. Besides its affordability, another advantage of ceramic membranes is that both its porosity and permeability can be customised by modifying the array of raw materials including: mullite, alumina, zirconia and other oxides [41], as well as by changing the firing cycles, curing time and temperature. Moreover, by customising the ceramics, an increased selectivity for cationic transfer can also be achieved [20].

By modifying the composition and firing cycles of the ceramic membranes, the pore distribution, permeability and water absorption will also vary, influencing the flux of ions and solvent through the membrane [41]. When an electric field is applied across an ion containing membrane, the electro-osmotic phenomenon takes place, where ions move through the membrane due to electromotive forces, accompanied by solvent molecules. The electroosmotic flow was first reported by F.F. Reuss who forced a water flux through a porous clay plug by applying an electric field [34]. A specific number of solvent molecules accompany each particular ion according to their electro-osmotic drag coefficient [31]. Due to the electro-osmosis phenomenon, together with the diffusion and osmotic pressure, a liquid electrolyte named catholyte is synthesised in the cathode chamber of ceramic MFCs whilst generating electricity [11,24].

Several parameters of the ceramic membrane play an important role in the catholyte collected. It has been reported that the thickness of the ceramic membrane has an effect on the amount and quality of the catholyte collected [24]. Other parameters including the pore size and the permeability of the ceramic part will also have an effect on the amount and quality of the catholyte solution formed in the cathode chamber. Consequently, the ORR mechanism and kinetics might also change, since the pH of the catholyte can be highly affected by the physicochemical parameters of the ceramic membrane [24,32]. The influence of the pH on the ORR will ultimately affect the overall fuel cell power generation [32]. Therefore, it is important to fully understand the effect of the pore size and the water absorption on catholyte quality and MFC power generation. Besides the catholyte generation, the separators highly contribute to the ultimate internal resistance of the system, which is a key factor in MFCs and in fuel cells in general, since it can affect the power generation [2,17]. The ohmic losses can be calculated from the contribution of the resistance to the ion transport through the electrolyte and the membrane, the resistance to the electron transport within the electrodes and current collectors, and the contact resistance [8]. The contribution of the ceramic membrane to the ohmic losses is directly related to its ionic conductivity. When using ceramic membranes, the ionic conductivity will be dependent on the type of clay and particle size of the raw material, which will affect the physical properties of the final product, such as dry strength, deformation, and water absorption [15,20,31,34].

Fireclay sanitaryware bodies generally consist of ball clay and have water absorption levels of approximately 9.0% in weight after firing. This value can indeed be modified by slightly changing the raw materials composition, as well as the firing process. Slow-firing cycles, which can reach up to 60 h, are typically used for manufacturing ceramics worldwide [9,36]. The water absorption and porosity have been reported to be higher in the fast-fired clays, while lower linear shrinkage and flexural strength were obtained after low-fired clays [26]. Regarding pore size distribution, larger pores were observed for calcareous clays in fast firing, whereas smaller pores in low firing were reported for non-calcareous clays [6]. Therefore, by modifying the structural parameters and the magnitude of firing cycles, the quality of the ceramic material can be adjusted [22]. However, it has also been reported that the water absorption in red clay decreases with increasing firing temperature, independently of the firing cycle [36]. Thus, it will be a combination of the speed firing cycle and the firing temperature that will affect the water absorption of ceramic membranes.

This work aims to understand the effect that the water absorption and the changes in the firing cycle of fine fire clay has in the ohmic resistance, the ionic conductivity and the power generated by the ceramic MFCs treating urine.

## 2 Materials and methods

### 2.1 Membrane characterisation

In order to study the effect that the water absorption of the fine fire clay cylinders has on power generation, a total of 6 different types of Fine Fire Clay (FFC) cylindrical samples (ROCA Sanitario S.A.) were tested as membrane and supporting material for microbial fuel cells treating urine. Three of the samples (GR GV) were manufactured using three different firing cycles, the first firing cycle (1 F) used a maximum firing temperature of 1206 °C, whereas the second firing cycle (2 F) reached 1205 °C and the third cycle (H.E.) was specifically adjusted to achieve a ceramic with higher water absorption, with a maximum temperature of 1150 °C. The remaining three types were manufactured adjusting the chemical composition of the fine fire clay (GR Modified) by decreasing the alkaline and earth concentration, with silicon as the main component. The same three different firing cycles 1 F, 2 F and H.E. were also used for the ceramics with modified composition. The images of the fine fire clay samples were captured using a Philips XL30 scanning electron 98 microscope (SEM). The water absorption was determined according to the EN 997:2012 regulation by measuring the mass difference between the dry membrane (*W*_*dry*_) and the wet membrane (*W*_*wet*_). The water absorption was then calculated using the following equation:

(1) $$WaterAbsorption\left(\%\right)=\frac{W_{wet}-W_{dry}}{W_{dry}}\times 100$$

### 2.2 MFC assembly and operation

**Fig. 1:**
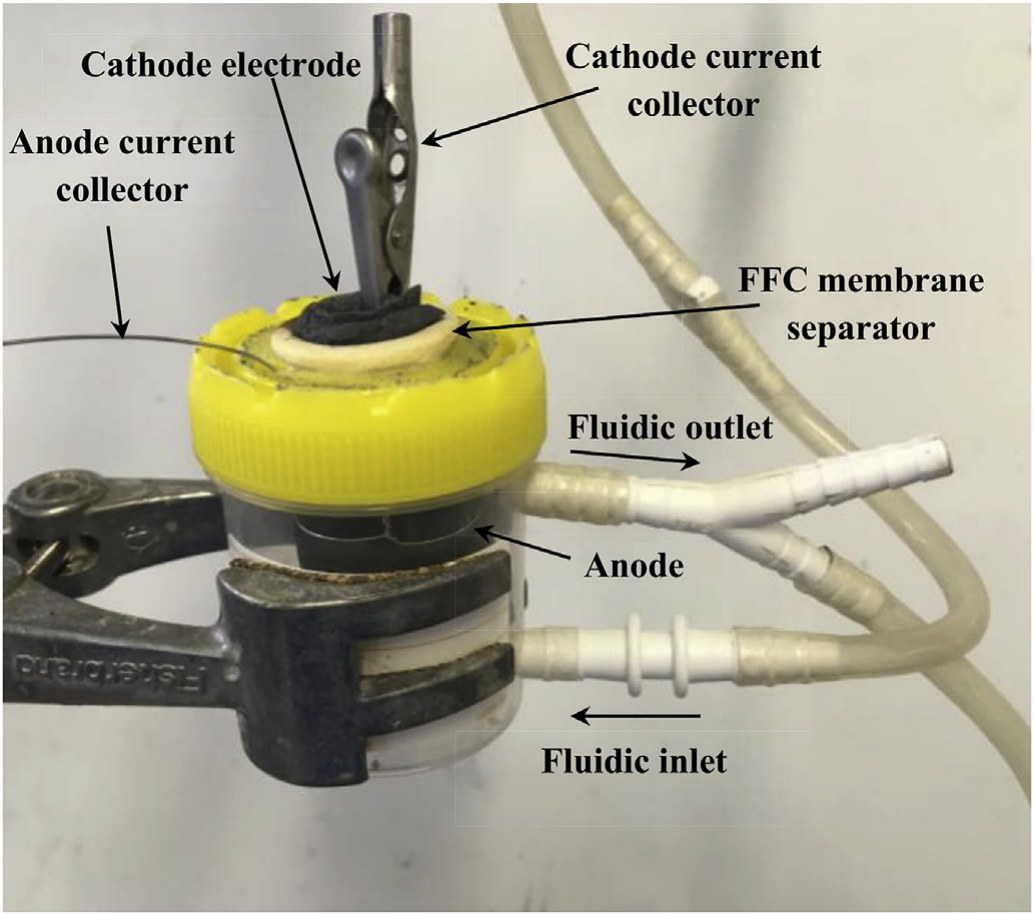
Picture of an MFC part of the experimental set up for power generation comparison.

A total of 18 MFCs were assembled to have triplicates of each type of fine fire clay ceramic MFCs. Ceramic cylinders of 5 mm thick, 23 mm out-diameter and 50 mm height were used. The MFCs were assembled with an anode outside-cathode inside configuration using a 270 cm^2^ piece of carbon veil (20 g/m^2^, PRF Composites, Dorset, UK) folded and wrapped around the ceramic cylinder. A stainless steel wire (0.5 mm, Scientific Wire Company) was also wrapped around the cylinder to hold the carbon veil anode electrode in place, as well as to be used as the current collector. The cathode electrodes were made as previously described [11] using a mixture of activated carbon (G. Baldwin & Co, 80 gr) and a polytetrafluoroethylene PTFE (Sigma-Aldrich) solution 30%wt. spread on a carbon veil support. The cathode electrodes were then cut with a geometric area of 25 cm^2^ and introduced into the internal part of the ceramic cylinders. A crocodile clip biting the cathode electrode was used as the current collector. The MFC assembly was then introduced in a plastic container with inlet and outlet connectors to allow continuous flow. The design allowed the cathode to be air-breathing and the cathode chamber to be empty of catholyte until it was formed during operation, whilst maintaining only the anode electrode in contact with the urine flow. The anolyte volume in the container was 20 mL. Fig. 1 shows the experimental set up. The MFCs were inoculated during three consecutive days with a mixture of 50% activated sewage sludge (Wessex Water Scientific Laboratory, Saltford, UK) and 50% urine provided by healthy individuals with a normal diet. The MFCs started operating under open circuit conditions for the first 2 h. Then, an external resistor of 1000 Ω was connected to each of the MFCs. After the third day, the MFCs were fed with 100% urine. After the inoculation phase, the continuous flow of 100% urine was set up to 200 mL day^−1^, which remained constant for the duration of the experiment, using a 16-channel peristaltic pump (205 U, Watson Marlow, Falmouth, UK). Single channels from the peristaltic pump were used for each MFC to achieve individual feeding directly from the urine bottle. The power generated was calculated using the Ohm's law, from external resistor value and the MFCs voltage, which was monitored using a data logging system (Agilent, KEYSIGHT, 34972 A LXI data acquisition/Switch unit).

In order to evaluate the optimum load and measure the maximum power, polarisation runs were performed periodically using a specially designed DR07 decade variable resistor box (ELC, France), which applied a range of external resistance values from 30 KΩ to 3.74 Ω for 5 min, to each MFC. All the MFCs were connected to equal external loads, whose value was adjusted to achieve maximum power generation from the most powerful MFCs, regardless of whether or not that specific load was the optimum for all types of MFCs. The cathode electrode voltage values were measured during the polarisation by adding a separate reference electrode in the cathode chamber (Dri-Ref, 2 mm diameter, World Precision Instruments, 30 mV *vs.* Ag/AgCl (1 M KCl, Sigma-Aldrich). The anode electrode voltage values (*V*_*a*_) were calculated from the MFC voltage and the cathode electrode voltage (*V*_*c*_) and using the following equation [32]:

(2) $$V_{MFC}=\left(V_{c}-V_{a}\right)-\sum lR$$

where *I* is the current generated by the MFCs and *R* is the combination of ohmic losses and the electrode and the electrolyte losses.

Catholyte samples were collected from all the MFCs at the end of the experiment. The pH and conductivity of the collected samples were measured using a Hanna 8424 pH meter a 470 Jenway conductivity meter (Camlab, UK), respectively. Urine and effluent samples were also collected and the chemical oxygen demand (COD) was analysed using the potassium dichromate oxidation method (COD HR test vials, Camlab, UK) with an MD 200 photometer (Lovibond, UK).

The coulombic efficiency (CE) was calculated according to the following equation [21]:

(3) $$C_{E}=\frac{M\cdot l}{F\cdot n\cdot q\cdot \Delta COD}$$

Where M is the molecular weight of the substrate (O_2_, 32 g mol^−1^), I (A) is the current generated from the MFC, F is the Faraday's constant (96485 C mol^−1^), n is the number of electrons exchanged per mol of oxygen during the ORR (4), q is the flow rate (L·s^−1^) and ΔCOD (g·L^−1^) is the COD difference between influent and effluent.

### 2.3 Impedance spectroscopy (IES)

The ionic conductivity of the fine fire clay ceramic membranes was determined by means of impedance measurements using a μAutoLab Type III with a frequency response analyser FRA 2. The frequency interval employed for the measurements ranged from 100 kHz to 10 mHz at AC amplitude of 10 mV. A conductivity homemade cell configuration was assembled using two H-Type glass bottles joined by a 3-D printed holder. Two electrodes, acting as working and counter electrodes, were sandwiching the membrane on both sides. An unused ceramic membrane disk (23 mm) of the same ceramic thickness as the cylindrical membranes (5 mm) was used. The reference electrode channel was connected to the counter electrode, since a two electrode configuration was used. The volume in each compartment was 200 mL of 100 mM NaCl. The conduction property of a material could be identified by extracting the bulk resistance (*R*_*b*_) from the Nyquist plot [33]. The ionic conductivity of each sample was calculated using the following equation:

(4) $$\sigma =\frac{L}{R_{b}A}$$

where *σ* is the ionic conductivity (S cm^−1^), *L* the thickness of the ceramic membrane (4 cm), *A* the contact area between the electrodes and the ceramic membrane (i.e., the electrode surface area, 1.33 cm^2^), and *Rb* the bulk membrane resistance (Ω) calculated from the Nyquist plot.

## 3 Results and discussion

Table 1 shows a comparison of the water absorption values for each type of cylinder manufactured under the three different firing cycles (1 F, 2 F and H.E.). For simplicity, names from FFC 1 to 6 have been assigned to each type of ceramic membrane.

**Table 1:** Water absorption of the 6 different fine fire clay cylindrical samples tested. The samples were obtained from two different compositions and three different firing cycles. In brackets the number assigned to each ceramic type for future references.

	Water absorption, %		
	Firing Cycle 1 F	Firing Cycle 2 F	Firing Cycle H.E.
Composition	Max. firing T^a^		
	1206 °C	1205 °C	1150 °C
GR GV	11.72 (FFC 1)	12.78 (FFC 2)	13.37 (FFC 3)
GR Modified	15.72 (FFC 4)	16.37 (FFC 6)	16.25 (FFC 5)

**Fig. 2:**
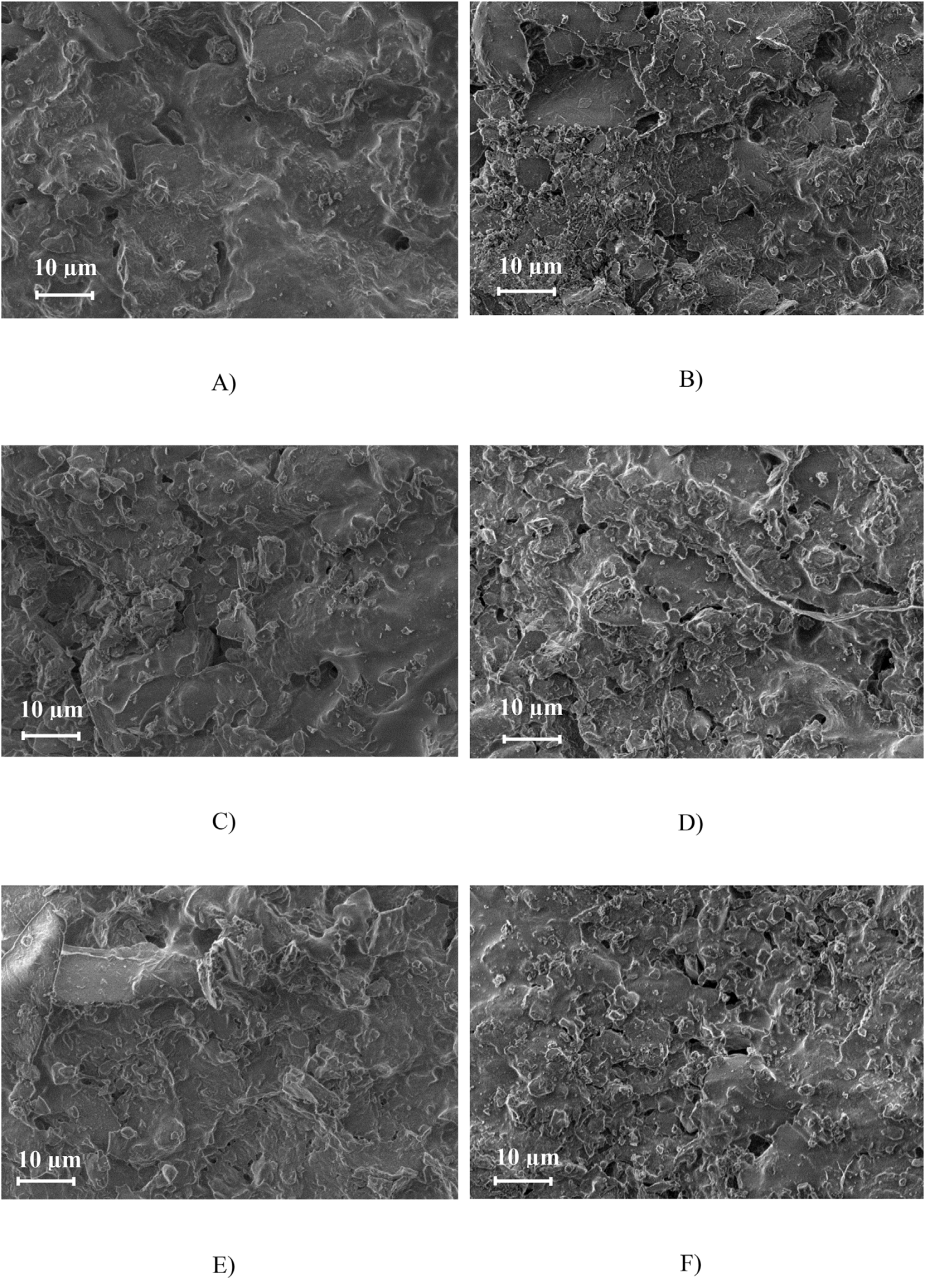
SEM pictures of the two types of FFC: GR GV and GR modified cured following three different firing cycles: 1 F, 2 F and H.E. A) FFC 1, B) FFC 2, C) FFC 3, D) FFC 4, E) FFC 5, F) FFC 6.

Fig. 2 shows the SEM images of the 6 different types of FFC tested. FFC 1 shows a higher number of smaller pores comparing it to FFC3, which shows a heterogeneous porous structure, with areas within the ceramic, where no pores are observed and other areas where large size pores of the order of 2–5 μm could be measured. FFC 2 showed a similar structure to FFC 1 with an apparently larger number of pores. FFC 4, 5 and 6 showed smaller ceramic granules, leading to a more porous structure. The pore sizes are not uniform with small pores from 200 nm up to larger pores of approximately 5 μm. FFC 5 and 6 have small pores (∼200 nm) as well as large pores (∼5  μm). However, more elongated pores and slightly larger were observed in FFC 6 compared to FFC5. FFC 4 also showed a wide pore distribution with sizes from 400 nm to ∼5  μm. Larger areas with no pores were also observed in FFC 4 compared to FFC 5 and 6.

**Fig. 3:**
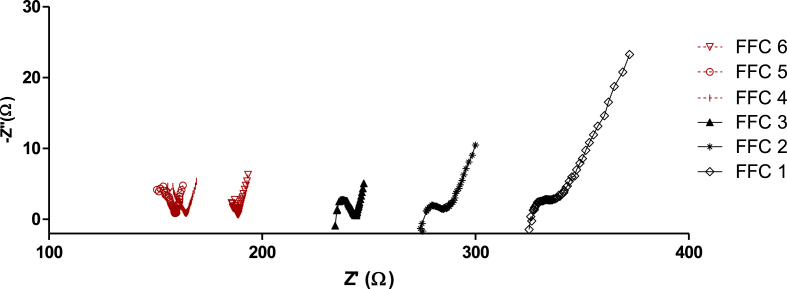
Nyquist plot obtained from the IES of the six FFC samples tested. In black are the samples with GR GV composition (FFC 1–3) and in red are the samples with modified composition (FFC 4–6). (For interpretation of the references to colour in this figure legend, the reader is referred to the Web version of this article.)

Fig. 3 shows the Nyquist plot from the IES performed with the different FFC samples tested in the MFCs. The difference in the ohmic resistance recorded for the different materials is noticeable, with a decreasing value for those materials with higher water absorption, as shown in Table 1.

In the Nyquist plot obtained from samples FFC 1, 2 and 3 the same shape is observed: a semi-circle in the high region and an inclined/straight line in the low frequency regions, noticing that the shape of the semicircle is depressed in nature from high to medium frequency region, which is an indication of micro-roughness and other inhomogeneities of the working electrode during the reaction [12]. An inclined line due to diffusion of the mobile species into the electrode material (mass transfer control area) is observed in all cases. This type of response is commonly observed in fuel cells and it is referred to as Warburg-type behaviour. The bulk resistance (*R*_*b*_) of each sample was given by the intersection of the semicircle with the real axis in the Nyquist plot [3,29]. In a standard Nyquist plot a complete semi-circle is usually obtained at the high frequency regions, as shown for samples FFC 1, 2 and 3. However, in the Nyquist plot of samples FFC 4, 5 and 6, an incomplete semicircle was observed. The reason for that can be related to the difference in structure and surface roughness between samples FFC 4, 5 and 6 compared to 1, 2 and 3, since they have slightly different composition, GR Modified vs GR GV, respectively [5,28,33]. The resulting data points for the bulk resistance (*R*_*b*_) of the samples FFC 4, 5 and 6, were calculated by graphical means given by the first intersection of the semicircle with the Z’ axis (high frequency region).

The ionic conductivity of each type of clay could then be calculated using equation (4) and is shown in Table 2. According to the data obtained, there is a correlation between the ionic conductivity and the water absorption of the clay. As it was expected, a more dense ceramic material, will pose a higher ohmic resistance. The ionic conductivity values reported herein are in the same order of magnitude as those previously reported, measuring ionic conductivity of ceramic materials using impedance spectroscopy [4,18,19,27]. FFC 5 is the ceramic material with the highest ionic conductivity. This might be related to the firing temperature, since it was fired at the highest temperature.

**Table 2:** Bulk resistance and ionic conductivity of each type of FFC.

Ceramic type	*R*_*bulk*_*(Ω)*	*σ* (S cm^−1^)
**FFC 1**	325.5	0.009
**FFC 2**	275.4	0.010
**FFC 3**	234.4	0.012
**FFC 4**	156	0.019
**FFC 5**	150	0.020
**FFC 6**	179.5	0.016

**Fig. 4:**
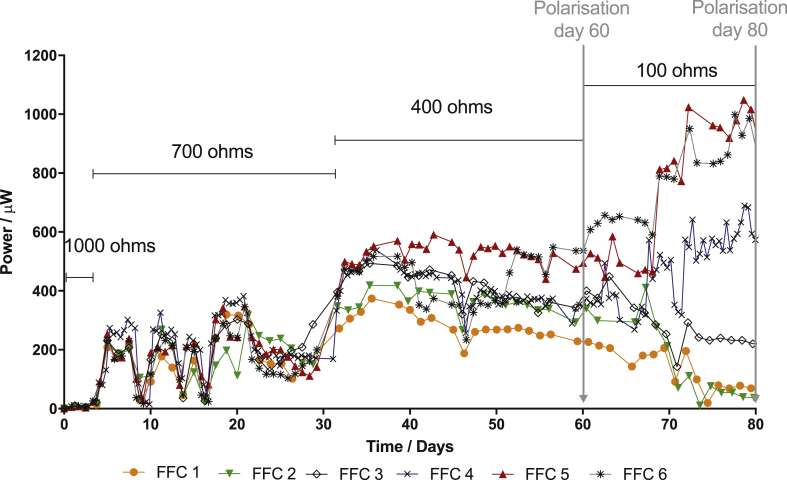
Average of the power monitored with time for the duration of the experiment for each type of ceramic MFCs: FFC 1 to 6. The average was calculated from triplicates of each type.

**Fig. 5:**
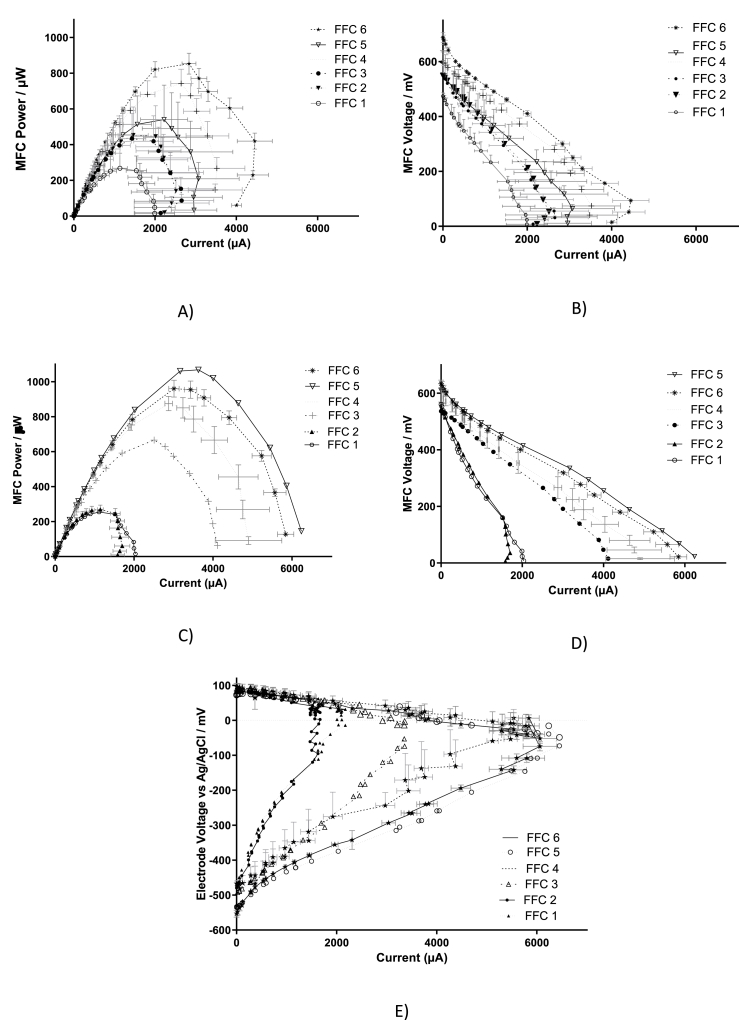
Polarisation performed after 60 days of operation: A) Power and B) Voltage of the MFC. Polarisation of the best performing MFCs after 80 days of operation: C) MFC power and D) MFC voltage, E) anode and cathode polarisation.

Fig. 4 shows the power generated from the ceramic MFCs assembled with FFC with different water absorption for the duration of the experiment. As shown in the figure, for the first 5 days all the MFCs generated the same power, however, when the value of the external resistor was changed to 700 Ω FFC 5 showed slightly higher power compared with the others. When the external resistors were changed to 400 Ω, the MFCs with the lowest water absorption ceramics, FFC 1 and 2, produced considerably lower power compared with the other MFCs. When 100 Ω was applied, there is a clear distinction between the ceramic types, showing a correlation between the water absorption of the ceramic and the MFC power output. The results from the polarisation performed after 60 days of operation is shown in Fig. 5, where again a correlation between the water absorption and the power generated from the MFCs can be clearly observed. As expected, the materials with higher ohmic losses, according to Table 2, led to a lower power generation. The difference in the ohmic losses can also be appreciated by comparing the polarisation potential of the MFCs tested. Moreover, these ceramic materials also posed higher resistance to the ion transfer, which also contributes to a lower power generation, including FFC 1, 2 and 3. The ceramic MFC assembled with FFC 4, 5 and 6 showed higher power output, demonstrating once again that higher power generation can be achieved from a material with higher water absorption and ionic conductivity. The optimum load according to this polarisation experiment was 100 Ω for FFC 4, 5 and 6.

Another polarisation was performed after 80 days of operation, shown in Fig. 5C–E, when all the MFCs had been working under an external load of 100 Ω for 20 days. A maximum power generation of 665 μW was obtained from FFC 3, which is 60% higher than that from FFC 1 and 2, but 24% lower that the power generated from FFC 4. The maximum power outputs were 960 and 1068 μW, generated from FFC 6 and 5, respectively. Fig. 5C and E and 5D suggest that FFC 1 and 2 were specially underperforming in comparison with the other MFCs. Fig. 5E shows a similar cathode performance in all MFCs but differences in the anodic performance, demonstrating the anodic nature of the differences in MFC power output. The anode polarisation, shown in Fig. 5E shows a clear difference in the anodic ohmic losses, especially between FFC 1 and 5, which is attributed to the ceramic materials and their differences in ion conductivity. Moreover, FFC 1 to 3 had been connected to a heavier load than their optimum, which caused a decrease in performance between day 60 and day 80. On the contrary, FFC 5 and 6 show good anodic performance, suggesting that the load applied (100 Ω) during the last period increased their optimum performance and that the ceramic membrane can cope with the ionic transfer.

The COD reduction was also calculated obtaining a correlation with the power output, with a maximum COD reduction of 15.5% for the FFC 5, followed by FFC 6 with 13% and FFC 4 with 9.5% per individual MFC. The MFCs with the FFC materials with lower water absorption led to a lower COD reduction with 6.2% for FFC 3 and lower than 5% for FFC 2 and 1. It should be considered that FFCs with FFC 1, 2 and 3 were underperforming since the systems were not operating under the optimum external load, but overloaded with a heavier load that was optimum for FFCs 4, 5 and 6. These COD values can be correlated with the current generated during operation, since current values of 3.3, 3.0 and 2.2 mA, were obtained from FFCs 5, 6 and 4, respectively; whereas 1.5, 0.9 and 0.7 mA were obtained from the FFCs 3, 2 and 1, respectively, for the final operation period. The coulombic efficiency values calculated for FCCs 5, 6 and 4 were 13.7%, 13.4% and 13.0%, respectively.

In summary, the results show that the MFCs using the modified composition of the fine fire clay obtained higher power generation than the ceramics with the original composition. This suggests that there is an increase in the power generated with the water absorption of the ceramic membrane. This trend is observed for both material compositions, suggesting that the water absorption is mostly causing the increase in the power generation of the GR Modified in comparison with the GR GV rather than the change in composition of the ceramic material.

**Fig. 6:**
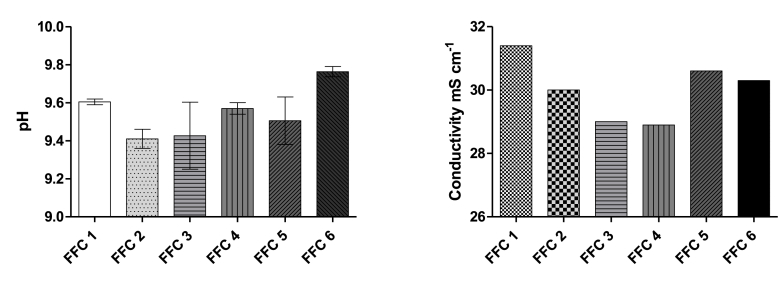
pH and average of the conductivity of the catholyte collected from the different type of ceramic MFCs.

Fig. 6 shows the pH and conductivity of the catholyte generated in each type of ceramic MFC. The pH from the catholyte solutions collected from the different MFCs varied from 9.4 to 9.8, whereas the conductivity varied from 29 to 31.5 mS cm^−1^. Previous studies focused on the catholyte generation from fine fire clay MFCs and the effect of the ceramic thickness in the catholyte quality [24]. The pH and conductivity shown in this work are in agreement with those previously reported [24,25], which showed a slight increase in pH and decrease in the conductivity with the power generated from MFCs of a ceramic thickness of 5 mm. In this study, the thickness of the ceramic MFCs was 4 mm and a slightly lower variation in pH was observed, when compared with the pH of urine, as expected. It has also been reported that thicker ceramic membranes, of the order of 10 mm, can produce higher quality of catholyte, highly alkaline and with bacterial killing properties [25]. The same work showed that lower power generation was obtained from the ceramic MFCs with the thicker membranes. However, by modifying the properties of the ceramic material to increase the water absorption, the power output of a thick ceramic MFC (10 mm) could be increased, as shown herewith, while producing high quality catholyte that can be used for practical applications, i.e. pathogen killing. Further work needs to be performed to assess the quality of a catholyte generated from a ceramic (10 mm thick) MFC with a high water absorption, above 16%. That would increase the catholyte quality, as well as, the power generation.

## 4 Conclusions

Fine fire clay commonly used in sanitaryware bodies, was used as a membrane and structural material for MFCs. A total of 6 types of FFC, with two compositions cured using three different firing cycles have been tested. These results demonstrate the effects of the physico-chemical properties of the ceramic membranes on MFC power generation and catholyte accumulation. The power output of the MFCs was increased by 64% only by changing the ceramic properties to higher water absorption. The FFC with higher water absorption showed lower ohmic resistance, whilst having higher ionic conductivity, leading to an increase in power output.
